# Point of care testing for the diagnosis of periprosthetic joint infections: a review

**DOI:** 10.1051/sicotj/2024019

**Published:** 2024-06-07

**Authors:** Pavlos Altsitzioglou, Konstantinos Avgerinos, Vasileios Karampikas, Panayiotis Gavriil, Apostolos Vlachos, Fotini Soucacou, Ioannis Zafiris, Vasileios Kontogeorgakos, Panayiotis J. Papagelopoulos, Andreas F. Mavrogenis

**Affiliations:** From the First Department of Orthopaedics, National and Kapodistrian University of Athens, School of Medicine Athens Greece

**Keywords:** PJI, Point of care testing, Alpha-defensin, Leukocyte esterase, Calprotectin, CRP

## Abstract

*Background*: Periprosthetic joint infection (PJI) remains a major complication following total joint arthroplasties (TJA), significantly affecting patient outcomes and healthcare costs. Despite advances in diagnostic techniques, challenges persist in accurately diagnosing PJI, underscoring the need for effective point-of-care testing (POCT). *Methods*: This review examines the current literature and latest developments in POCT for diagnosing PJI, focusing on biomarkers such as alpha-defensin, leukocyte esterase, calprotectin, and C-reactive protein (CRP). Criteria from various societies like the Musculoskeletal Infection Society, Infectious Diseases Society of America, and the International Consensus Meeting were compared to evaluate the effectiveness of these biomarkers in a point-of-care setting. *Results*: POCT provides rapid results essential for the timely management of PJI, with alpha-defensin and leukocyte esterase showing high specificity and sensitivity. Recent advancements have introduced novel biomarkers like calprotectin, which demonstrate high diagnostic accuracy. However, challenges such as the variability in test performance and the need for validation under different clinical scenarios remain. *Discussion*: While POCT for PJI shows promising results, their integration into clinical practice requires standardized protocols and further validation. The evolution of these diagnostic tools offers a potential shift toward more personalized and immediate care, potentially improving outcomes for patients undergoing TJA.

## Introduction

Periprosthetic joint infection (PJI) is a major complication of total joint arthroplasties (TJA). Despite receiving the best available treatment, PJI can substantially diminish a patient’s quality of life and increase patient mortality [1]. According to Kurtz et al. [2], the risk of PJI within one year after total hip arthroplasty (THA) is 0.7%, and the risk within five years is 1.1%. For total knee arthroplasty (TKA), the corresponding risks are 0.7% within one year and 1.4% within five years. The one-year and five-year survival rates after the diagnosis of PJI were 88.7% and 67.2% for THA and 91.7% and 71.7% for TKA, respectively. The majority of revision TJA surgeries are conducted to address PJI and account for 25% of cases [3].

The management of infected and non-infected failures following TJA varies greatly and can have a substantial impact on patients [4]. Hence, it is crucial to refrain from mistaking a healthy joint for an infected one, and vice versa, as doing so can result in higher rates of illness, unnecessary expenses, and preventable surgical procedures [5]. Precise and reliable diagnostics are extremely important in clinical practice to ensure proper treatment and prevent these negative consequences [6]. Diagnosing PJI can be straightforward in certain situations, where clear clinical evidence such as the existence of a sinus tract or pus around the prosthesis are regarded as definitive diagnostic criteria [7, 8]. In contrast, the absence of these confirming criteria in numerous instances poses a significant challenge in diagnosing PJI [9]. In these cases, the diagnosis usually depends on laboratory tests, including serology or analysis of synovial fluid, microbiological analysis of tissue specimens or synovial fluid, as well as histological and radiographic findings.

Recently, efforts have been made to enhance the precision of diagnostic procedures. In 2011, the Musculoskeletal Infection Society (MSIS) categorized PJI “major” criteria (existence of a communicating sinus tract and two positive periprosthetic cultures), and “minor” criteria (increased ESR/CRP levels, synovial leukocyte count, and synovial polymorphonuclear cell levels, presence of purulent material, isolation of a single organism in a culture, and intraoperative frozen sections with histology) [10]. In 2013, the Infectious Diseases Society of America (IDSA) established its own diagnostic criteria for PJI in order to standardize the diagnostic process [11]. The IDSA criteria differ from the MSIS criteria in that they do not take into account elevated inflammatory markers. Instead, the IDSA criteria consider other factors such as the presence of a virulent organism from a single culture or the presence of acute inflammation from histopathology of the periprosthetic tissue. In 2013, the International Consensus Meeting (ICM) implemented a novel minor criterion, which involved measuring the leukocyte esterase in synovial fluid using a urine strip test. In 2018, Parvizi et al. [7] enhanced the ICM concept by introducing a scoring system that takes into account the varying sensitivity and specificity of biomarkers. The updated system incorporated promising novel indicators, such as alpha-defensin in synovial fluid and D-dimer in serum. The European Bone and Joint Infection Society (EBJIS) criteria were implemented in 2021 to categorize cases as “unlikely,” “likely,” or “confirmed”; in these cases, the diagnosis is established by analyzing clinical, laboratory, microbiological, and histological data, as well as intraoperative findings (Table 1).

**Table 1:** Summary of the diagnostic criteria for PJI according to various guidelines from 2011 to 2021. Criteria include sinus tract formation and purulent material, laboratory biomarkers such as CRP and ESR, synovial fluid analysis, and other diagnostic methods such as microbiology and histology.

Diagnostic criteria	MSIS 2011	IDSA 2013	ICM 2013	ICM 2018	EBJIS 2021
Clinical					
Sinus tract	Yes	Yes	Yes	Yes	Yes
Purulent material	Yes	Yes	No	Yes	Yes
Blood					
CRP (mg/L)	High	No	10	10	>10
ESR (mm/h)	High	No	30	30	No
D-Dimer (μg/L)		No	No	30	No
Synovial fluid cytological analysis					
Synovial leukocyte count (cells/uL)	Yes	No	3000	3000	>1500
Synovial PMN (%)	High	No	90	70	65
Synovial fluid biomarkers					
Alpha defensin	No	No	No	1.0	Yes
Leukocyte esterase	No	No	Positive	Positive (high)	No
Microbiology					
Culture	1	1	Yes	Yes	>1
Sonication (CFU/ml)	No	No	No	No	>1
Histology					
High-power field (400x magnification)	>5 neutrophils per HPF in 5 PHF	Yes	>5 neutrophils per HPF in 5 PHF	Yes	>5 neutrophils in single HPF
Other					
Nuclear imaging (WBC scintigraphy)	No	No	No	No	Yes

CRP: C-reactive protein; ESR: erythrocyte sedimentation rate; PMN: polymorphonuclear neutrophils; WBC: white blood cell count.

Although there have been advancements in recent years that have increased the chances of correctly diagnosing PJI by introducing various PJI criteria, the discovery of novel biomarkers that are both highly specific and sensitive for PJI, particularly in the form of point of care testing (POCT), could facilitate a simpler and more precise diagnosis of this substantial complication. At present, the four most prevalent biomarkers available in this respect are alpha-defensin, leukocyte esterase, calprotectin, and CRP. These biomarkers enable the clinician to obtain immediate results without the necessity of sending the patient’s synovial fluid to a laboratory for testing. This review aims to gather and assess all the existing information regarding these innovative POC biomarkers for the preoperative diagnosis of PJI.

## Point-of-care testing

POCT is the practice of conducting laboratory tests in close proximity to patients. Typically, POCT involves laboratory analysis that is conducted outside of the laboratory. This type of testing does not require any sample preparation or pipetting steps. It uses pre-made reagents that do not need to be modified by the operator. Additionally, POCT provides immediate indications for potential therapeutic approaches based on the test results. Undoubtedly, these analyses are highly efficient and straightforward to interpret [12]. Certain POCT methods provide precise and dependable results promptly, resulting in a notable time benefit when making critical decisions about diagnostic and therapeutic procedures.

POCT devices in Europe are subject to regulation according to the European Directive 98/79/EC on in vitro diagnostic medical devices, which was established in 1998 [13]. Generally, in vitro diagnostic assays, such as POCT, can be sold if they have successfully undergone a conformity assessment procedure and have been granted a CE mark. This mark indicates that the product complies with the European directives for *in vitro* diagnostic testing (IVD Directive) [12]. The test manufacturer is responsible for conducting tests and providing data on the device’s performance. The user’s responsibility is to assess whether the POCT is appropriate for its diagnostic purpose and, therefore, beneficial for clinical application. Nevertheless, in order to guarantee the effectiveness and adherence to regulations of POCT, various strategies have developed in recent years. Representatives from nursing and laboratory staff, along with a medical director, administer POCT programs in many hospitals [14]. In addition, effective management of POCT requires laboratories to conduct regular inspections to ensure compliance with regulations and maintain a consistent supply of specialized staff in hospitals.

Various criteria, including medical, economic, and organizational factors, are considered when determining the activation of POCT. However, particular emphasis is placed on turnaround time (TAT) [15]. Specific parameters that provide crucial information about vital functions and influence the choice of therapeutic intervention, particularly in critical conditions, require a moderate TAT [15]. It is crucial to remember that the introduction of POCT enabled the performance of high-quality laboratory diagnostic tests by individuals who lack the required expertise in medical and laboratory technology. Hence, it is not advisable to use POCT assays in an unskilled and uncritical manner. Most importantly, these methods cannot replace the knowledge and proficiency of a medical laboratory.

The primary purpose of POCT is to aid in clinical decision-making. However, there is currently insufficient evidence to ascertain whether these devices can enhance patient management and decrease hospital admissions [16]. Furthermore, the absence of skilled laboratory personnel to analyze the results of tests may lead surgeons to inaccurate diagnoses, resulting in patients undergoing unnecessary surgical procedures.

## Alpha-defensin

Defensins are naturally occurring peptides that have the ability to kill microorganisms. They are effective against viruses with an outer envelope, fungi, and Gram-negative and Gram-positive bacteria [17]. They are able to eliminate pathogenic microorganisms by either creating pore-like openings in the cell membrane or by attaching to and enveloping the microbial membrane, causing breakdown and destruction of the microorganisms [18]. Alpha-defensins, specifically, are highly prevalent in neutrophils and macrophage populations. They are primarily released by polymorphonuclear cells as a response to pathogens [18]. Typically, α-defensin levels in synovial fluid are determined using an enzyme-linked immunosorbent assay (ELISA) that is specifically designed for this type of sample. This assay is optimized to eliminate any effects of varying viscosity between the samples being analyzed.

Alpha-defensin production is regulated by several pro-inflammatory cytokines, including interleukin-1β (IL-1β), interleukin-6 (IL-6), and tumor necrosis factor-α (TNF-α). These cytokines have the ability to increase the expression of alpha-defensin [19]. Interestingly, the levels of alpha-defensin do not appear to be influenced by the use of antibiotics for treating PJI prior to diagnostic evaluation. There has been no observed decrease in alpha-defensin levels following the administration of antimicrobial drugs [20]. On the other hand, antibiotic treatment significantly decreases the levels of CRP, white blood cells, and polymorphonuclear cell counts. This can cause misleading outcomes and an inaccurate diagnosis of PJI [20] Additionally, the alpha-defensin ELISA test has been recognized for its high sensitivity and specificity (>95%) in the diagnosis of THA and TKA PJI [21]. Moreover, the synovial fluid alpha-defensin immunoassay was found to be similar to the test that assesses leucocyte esterase, an enzyme released by neutrophils in infected joint fluids [22].

The alpha-defensin immunoassay is simpler and more effective than the MSIS criteria, which can be complex and potentially confusing [23]. This makes the alpha-defensin immunoassay an appealing tool for fast and accurate diagnosis of PJI. Indeed, the examination of alpha-defensin levels can serve as a valuable method to optimize the surgical approach, therefore, enabling the physician to promptly initiate an appropriate antibiotic treatment. However, Bonanzinga et al. [24] showed that although both positive (PPV) and negative (NPV) predictive values are elevated, only negative results can be regarded as predictive in the diagnosis of PJI. If the alpha-defensin immunoassay yields a negative result, it is highly probable that there is no PJI present. Conversely, if the result is positive, there is a strong likelihood of PJI. However, the increased level of alpha-defensin may be attributed to factors other than a periprosthetic infection. Metallosis can cause the alpha-defensin test to produce a false-positive result. This can be a misleading factor when interpreting alpha-defensin results and may lead to an incorrect diagnosis. In addition, Bingham et al. [25] observed increased levels of alpha-defensin along with CRP, ESR, and white blood cell count in two patients who did not have PJI. These authors regarded the possibility that aseptic inflammation could be the cause of the elevated alpha-defensin levels in these patients [25].

Therefore, it is crucial to utilize other precise diagnostic criteria for PJI in order to effectively manage patients who have undergone THA or TKA, or those who are suspected of having a PJI. The alpha-defensin immunoassay can be combined with PJI microbiological and biochemical tests to yield strong and dependable results. However, it should not be solely relied upon by orthopedic surgeons for the diagnosis of an ongoing PJI.

### Alpha-defensin POCT

Today, orthopedic surgeons are directly presented with a POCT assay (Synovasure, Zimmer, Warsaw, IN, USA) that can identify the presence of human alpha-defensin 1–3 in the synovial fluid of patients who are experiencing pain and/or inflammation in a TJA. The “alpha-defensin lateral flow” test is a visual immunochromatographic assay that consists of a single-use device, a premeasured vial of dilution buffer, a disposable Microsafe tube, and a sample cup [23]. The synovial fluid is appropriately diluted and introduced into the test device, where it moves towards the buffering pad and interacts with a gold conjugate that is labeled with an anti-defensin antibody. Ultimately, the combination moves from one end to another, passing through both the test line and the control line. After a duration of 10 minutes, the outcome is presented to the operator [26]. The assay yields only two potential outcomes: when the concentration of alpha-defensin exceeds the predetermined threshold, a test result line (“*T*”) will appear on the device, along with a reddish-pink control line (*C*-line); in the case of a negative result, only the reddish-pink control line will be visible on the device. Remarkably, this α-defensin immunoassay consistently yields results that are not influenced by the specific microorganism causing a PJI. Instead, it solely detects the presence of an ongoing infection [25, 27].

A recent study emphasized the wide range of microorganisms that are accountable for the secretion of alpha-defensin in synovial fluid, which is then identified through the alpha-defensin immunoassay [21]. However, there is currently no data available on the performance of this assay in the immediate postoperative period for severely immunocompromised patients or individuals with severe inflammation unrelated to PJI. In addition, Kasparek et al. [26] emphasized that the alpha-defensin intraoperative lateral flow test is comparable to a diagnosis based on the MSIS criteria and is highly valuable in confirming the absence of PJI. Nevertheless, the authors assessed the performance of an alpha-defensin POCT directly in the operating room, revealing a sensitivity of 67% and a specificity of 93%.

A perfect predictor would be characterized as having 100% sensitivity and specificity. However, it is important to acknowledge that all predictors are prone to some degree of error. Tests with sensitivity and specificity values >90% are considered highly credible for practical reasons. The data presented by Kasparek et al. [26] emphasize that a POCT assay with low sensitivity alone cannot be relied upon for an accurate diagnosis of prosthetic joint infection (PJI).

Frangiamore et al. [27] made the interesting observation that an alpha-defensin POCT demonstrated a high level of sensitivity and specificity (>90%) for first-stage and single-stage revisions. However, the test exhibited lower performance in the case of a second-stage revision. Specifically, the sensitivity decreased to 67%, while the specificity remained nearly unchanged. In addition, the authors did not conduct the alpha-defensin test in the operating room during surgery. Instead, they obtained synovial fluid samples and sent them to an external laboratory for analysis using the Synovasure test. Therefore, it is not possible to make any accurate assessment of the in-situ performance of the alpha-defensin POCT.

In order to guarantee accurate and skilled testing, it is recommended that for every ten POCT assays conducted in the operating room, one sample should be tested simultaneously and compared with the alpha-defensin ELISA in the central laboratory. A major issue with POCT is that numerous orthopedic surgeons lack sufficient experience and training in adhering to high laboratory standards. As a result, they often fail to recognize the importance of quality control and accurate documentation when employing POCT assays [15]. When taking all these factors into account, it becomes evident that the alpha-defensin assay, although a useful tool for orthopedics, should not be relied upon as the sole indicator to exclude PJI. Instead, it should be combined with the other MSIS criteria to achieve a more precise and accurate diagnosis.

## Leukocyte esterase

The International Consensus Meeting (ICM) took place in Philadelphia (USA) in 2018 and established the ICM 2018 International Consensus on Prosthetic Joint Infections [28]. In comparison to the widely utilized MSIS 2014 diagnostic criteria, the updated version remains fundamentally unaltered, with the exception of the addition of two primary diagnostic indicators. Furthermore, the secondary diagnostic indicator has been divided into four components: serological examination, synovial fluid analysis, microbial culture, and intraoperative indicators. When analyzing synovial fluid, the leukocyte esterase (LE) strip test is used along with leukocyte count and alpha-defensin detection. This modification carries significant importance in the updated edition of the PJI diagnostic criteria [7].

The LE strip test employs a plastic strip that has filter paper attached to one end, which contains indolyl carboxylate. LE catalyzes the conversion of the substrate into indole groups that are subsequently oxidized in the indoor air, resulting in the production of an indigo color. The presence of LE activity in body fluids was qualitatively determined by comparing the color of the strip with the colorimetric card during the LE strip test [29]. The LE strip test was initially used for the quick screening and diagnosis of urinary tract infections [30]. Subsequently, it has been extensively utilized in the areas of the digestive system [31], gynecological system [32], nervous system [33], and otolaryngology system [34]. The device is capable of detecting various bodily fluids, including ascites, gynecological secretions, cerebrospinal fluids, and sputum. The assay is an integral component of the systematic process used to screen for and diagnose infectious diseases.

Parvizi et al. [35] were the pioneers in utilizing the LE strip test for the diagnosis of PJI and incorporating it into the diagnostic framework for infectious diseases affecting the bone and joint system. Subsequently, scientists discovered that the LE strip test had a combined sensitivity and specificity of 93.3% and 77.0%, respectively, for the diagnosis of PJI. This was determined by using positive cultures or the presence of a draining sinus tract as the gold standard. The diagnostic tool is known for its fast speed, cost-effectiveness, and high sensitivity [36]. The LE strip test is an efficient method for the initial screening of infections. It has the ability to reduce costs and shorten the time required, resulting in significant savings in medical resources. Nevertheless, the strip test is susceptible to the subjective assessment of the tester, external environmental factors, and sample contamination [37].

### LE diagnostic accuracy

Several studies have demonstrated the validity and reliability of the LE strip test for the diagnosis of PJI. A recent meta-analysis by Chen et al. [38] incorporated 12 studies that utilized LE strip as a diagnostic tool for PJI [38]. The study’s findings revealed that the LE strip test had a combined sensitivity of 87% (95%CI, 84–90%) and a specificity of 96% (95%CI, 95–97%). The odds ratio (OR) was 170.09 (95%CI, 97.63–296.32). The LE strip test had superior performance compared to other serological and synovial fluid markers. For example, its sensitivity is 86% (95%CI, 82.5–89%) and specificity is 72.3% (95%CI, 70.4–74.2%), surpassing the performance of ESR. Similarly, the sensitivity and specificity of the LE strip test outperform synovial fluid procalcitonin (sensitivity, 53%; specificity, 92%), synovial fluid IL-6 (sensitivity, 72%; specificity, 91%), and synovial fluid CRP (sensitivity, 92%; specificity, 90%) (Table 2).

**Table 2:** Comparison of the sensitivity and specificity of the LE POCT with other commonly used infection biomarkers for the diagnosis of PJI.

Study	Parvizi et al. [35]	Wetters et al. [36]	Shahi et al. [37]	Chen et al. [38]	Aalirezaie et al. [39]	Carli et al. [40]	Kheir et al. [41]	Aggarwal et al. [42]	Li et al. [43]	Li et al. [44]
Aim	Determine sensitivity and specificity of leukocyte esterase in diagnosing PJI	Evaluate the diagnostic accuracy of leukocyte esterase reagent strips for diagnosing PJI	Evaluate effects of antibiotic administration on synovial leukocyte esterase strip test for PJI	Assess the diagnostic effectiveness of synovial fluid α-defensin and LE for PJI.	Analyze the diagnostic accuracy of LE strip test for PJI	Compare diagnostic accuracy of various tests for chronic PJI	Determine if LE is a good predictor of persistent infection and/or subsequent failure	Describe a simple, inexpensive, and effective protocol for using centrifugation with LE testing	Investigate the reliability of the LE strip test	Assess the impact of centrifugation on LE strip test results
Methods	Prospective study, preoperative and intraoperative synovial fluid analysis	Evaluation of 223 total hip or knee arthroplasties using leukocyte esterase reagent (LER) strips	Retrospective analysis of patients undergoing revision hip or knee arthroplasty	Systematic review and meta-analysis of studies assessing synovial fluid biomarkers for PJI diagnosis	Studies on synovial fluid α-defensin and LE for PJI diagnosis: meta-analysis	Systematic Cochrane review and meta-analysis of chronic PJI diagnostic tests	Prospective analysis of patients undergoing two-stage exchange treatment of PJI	Description of a protocol for using centrifugation with LE testing	Analysis of synovial fluid extracted by joint aspiration applied to LE strips	Analysis of LE strip test results before and after centrifugation
Results	++ reading had 80.6% sensitivity, 100% specificity for PJI diagnosis	Sensitivity: 92.9%, Specificity: 88.8%	Antibiotic administration led to decreased sensitivity of standard tests for PJI diagnosis	LE strip and α-defensin both have high sensitivity and specificity for PJI diagnosis	LE strip test had sensitivity of 85.7%, specificity of 94.4% for PJI diagnosis	Synovial α-defensin tests and LER strips had best performance for diagnosing chronic PJI	LE test sensitivity: 26.3%, Specificity: 100%; MSIS criteria sensitivity: 25.0%, Specificity: 87.3%	LE testing maintained accuracy after centrifugation	Sensitivity: 92.0% (500 threshold), Specificity: 93.1% (500 threshold)	Sensitivity: 97.7% before centrifugation, 92.5% after centrifugation
Conclusion	LE esterase in synovial fluid valuable for diagnosing PJI	LER strips rapid, inexpensive, sensitive tool for PJI diagnosis	Antibiotic administration interferes with standard diagnostic tests for PJI diagnosis	LE strip and α-defensin provide rapid and convenient diagnosis for PJI	LE strip test provides rapid, reliable diagnosis for PJI	Synovial fluid-based tests perform well for diagnosing chronic PJI	LE test may be indicative of persistence of infection, higher rate of subsequent failure	LE testing is reliable for diagnosing PJI, even with prior antibiotic administration	LE strip test is an accurate marker for diagnosing PJI	LE strip test results can be influenced by centrifugation
Pros	Real-time results, high sensitivity and specificity	Rapid, inexpensive, sensitive tool	Provides insight into effects of antibiotics on diagnostic tests, highlights importance of LE strip test	Rapid and convenient diagnosis, high sensitivity and specificity	Reliable diagnostic tool, high sensitivity and specificity	Perform well for diagnosing chronic PJI, provides comprehensive overview	Provides insight into predictor of infection persistence, helps in subsequent failure prediction	Simple and inexpensive protocol, effective in maintaining accuracy	Excellent sensitivity and specificity, reliable diagnostic tool	Overcomes interference from erythrocytes, maintains sensitivity and specificity
Cons	Limited to synovial leukocyte esterase, requires further validation	Limited to cases where synovial fluid is obtained, readability may be affected by blood or debris	Retrospective design, potential bias	Limited to synovial fluid biomarkers, requires further validation	Limited to LE strip test, may not account for all diagnostic markers	Limited to chronic PJI, potential bias in included studies	Limited to two-stage exchange treatment, may not generalize to other treatments	May not generalize to all scenarios, requires validation in broader contexts	Limited to LE strip test, may not account for other diagnostic markers	Influence of centrifugation on results requires consideration

A preliminary systematic review by Aalirezaie et al. [39] analyzed 11 primary studies that included 2061 patients. The findings indicated that the LE strip test had a sensitivity of 85.7% (95%CI, 65.9–90.7%) for the diagnosis of PJI. The specificity was 94.4% (95%CI, 85.3–97.7%), the PPV was 84.3% (95%CI, 71.5–91.7%), and the NPV was 94.0% (95%CI, 85.8–97.1%). Carli et al. [40] performed a comprehensive analysis of 203 studies. These studies assessed the serological, synovial, and histological markers in each diagnostic guideline for PJI. Their findings indicated that the laboratory tests for synovial alpha-defensin (ELISA) and LE strip test had the highest performance, followed by leukocyte count, synovial tissue CRP, PMN (%), and alpha-defensin assay. The Youden index ranged from 0.78 to 0.94; the Youden index for the three examinations (IL-6, CRP, and ESR) ranged from 0.61 to 0.75.

### LE limitations

The LE strip test demonstrates exceptional diagnostic efficacy and can be utilized effectively either on its own or in conjunction with other diagnostic biomarkers. It serves as a rapid screening tool and can also be employed to confirm the presence of a suspicious joint infection near the prosthesis. Nevertheless, certain glaring constraints must not be overlooked. One constraint is the issue of sample mingling; the presence of unwanted substances, such as blood, in the sample can lead to severe mixing, making the results of the LE strip test unreadable [41]. Centrifugation has been shown to be a viable solution in this respect [42]. Li et al. [43] showed that the sensitivity and specificity of the LE strip test remained consistent before and after synovial fluid centrifugation, indicating that centrifugation was a dependable procedure. In contrast, the findings of another study [44] showed that centrifugation can partially deteriorate the outcomes of the LE strip test. Currently, centrifugation is the sole available solution to address the issue of sample mingling. However, it has the potential to impact the final test outcomes. The matter continues to be a subject of debate and necessitates additional investigation. Moreover, there are doubts about the accuracy of the detection method and the reliability of the qualitative results obtained from the colorimetric comparison of LE strips [45]. The optimal quantity of synovial fluid samples needed for the LE strips test, as well as the appropriate timing for reading the results, have not yet been established by clinical studies. These areas are currently being investigated in further research [46].

LE POCT is a rapid and uncomplicated medical examination that can be conducted at the patient’s bedside to promptly provide the test result to the attending physician [47]. The limitations of the LE strip test are evident when considering its role as a basic diagnostic tool. Further research and development are needed for standardized operating procedures, which should include consistent time and sample size. Additionally, homogeneous test strip materials and supporting equipment suitable for joint fluid and other specific body fluids also require further investigation.

## Calprotectin

Calprotectin is a protein complex that is released during inflammation. It constitutes 60% of all soluble proteins found in neutrophils [48]. Neutrophils are attracted to areas where there is inflammation and infection; therefore, it is anticipated to observe elevated levels of neutrophil biomarkers in samples taken from infected patients [49]. Calprotectin is commonly employed as a diagnostic tool for inflammatory bowel disease and has demonstrated its ability to identify relapse in rheumatoid arthritis [50]. A recent study utilized a stool calprotectin test for off-label diagnosis of PJI, revealing an NPV of 94.4% [51]. This highlights the usefulness of calprotectin in the diagnosis of PJI and emphasizes the requirement for a verified lateral flow test.

Lyfstone AS, a company based in Tromsø, Norway, has recently created a lateral flow calprotectin test for the diagnosis of PJI. This test has successfully undergone the European in vitro diagnostic (IVD) regulatory approval process (98/79/EC). In 2020, Troter et al. [52] performed a preliminary investigation where they collected 69 synovial fluid samples from patients undergoing revision surgery at the Norfolk and Norwich University Hospital. The samples were obtained during the operation and were subsequently frozen. Retrospectively, synovial fluid calprotectin levels were assessed using a newly available lateral flow assay, named Lyfstone As, for the diagnosis of PJI. These results were then compared to the gold standards of the International Consensus Meeting (IcM) 2018 criteria and clinical case review (IcM-cR). Based on the IcM analysis, 24 patients were identified as positive for PJI, while the remaining 45 patients were negative. The lateral flow test showed an overall accuracy of 75.36% (52/69 patients; 95%CI, 63.51–84.95%) when compared to IcM. The sensitivity and specificity were 75.00% (18/24 patients; 95%CI, 53.29–90.23%) and 75.56% (34/45 patients; 95%CI, 60.46–87.12%), respectively. The PPV was 62.07% (18/29 patients; 95%CI, 48.23–74.19%) and the NPV was 85.00% (34/40 patients; 95%CI, 73.54–92.04%). The area under the receiver operating characteristic (ROC) curve (AUC) was 0.78 (95%CI, 0.66–0.87). The clinical team reviewed patient data from cases that showed disagreement in order to create the IcM-cR gold standard.

The performance of the lateral flow test showed a significant improvement compared to IcM-cR. The accuracy increased to 82.61% (57/69 patients; 95%CI, 71.59–90.68%). The sensitivity increased to 94.74% (18/19 patients; 95%CI, 73.97–99.87%). The NPV increased to 97.50% (39/40 patients; 95%CI, 85.20–99.62%). Additionally, the AUC increased to 0.91 (95%CI, 0.81–0.96). The test performance was superior in knees, achieving a 100.00% accuracy rate (17/17 patients; 95%CI, 80.49–100.00%), compared to hips, which achieved a 76.92% accuracy rate (40/52 patients; 95%CI, 63.16–87.47%).

In 2022, Waren et al. [53] conducted a study where they collected 123 samples of synovial fluid from patients undergoing revision TKA. These samples were then tested using a calprotectin lateral flow POCT assay. The data were examined and evaluated by two independent reviewers who were unaware of the calprotectin test results. The calprotectin lateral flow POCT showed exceptional sensitivity and specificity when evaluated against existing definitions for PJI. According to the 2013 Musculoskeletal Infection Society criteria, the calprotectin POCT showed a sensitivity of 98.1%, specificity of 95.7%, PPV of 94.5%, NPV of 98.5%, and an AUC of 0.969. The 2018 ICM was utilized to conduct the POCT, which yielded a sensitivity of 98.2%, specificity of 98.5%, PPV of 98.2%, NPV of 98.5%, and an AUC of 0.984. The POCT evaluated according to the 2019 proposed criteria by EBJIS showed a sensitivity of 93.2%, specificity of 100.0%, PPV of 100.0%, NPV of 94.2%, and an AUC of 0.966.

In the same year, Lazic et al. [54] conducted another prospective cohort study involving 33 patients. Out of these patients, 17 patients had undergone surgery within the past 9 months. Among these patients, 11 patients experienced dislocation and 5 patients experienced implant breakage. Analyzed were the synovial white blood cell count (WBC), percentage of polymorphonuclear neutrophils (PMC), serum CRP, and synovial calprotectin using a lateral-flow-assay. The parameters underwent testing using a modified EBJIS definition, with thresholds adjusted to accommodate the local inflammation. The statistical quality criteria were computed and compared using a binary classification test. A total of 17 patients were categorized as confirmed infections based on the modified EBJIS definition, with 13 patients involving THA and 4 patients involving TKA. The calprotectin assay showed a sensitivity of 0.88 (0.64, 0.99), a specificity of 0.81 (0.54, 0.96), a PPV of 0.83 (0.59, 0.96), and a NPV of 0.87 (0.60, 0.98). These results indicate that calprotectin is a dependable diagnostic parameter for detecting a PJI in both primary and revision THA and TKA, even in the presence of local inflammation caused by non-infectious factors.

Several meta-analyses have shown the diagnostic accuracy of calprotectin in detecting PJI. An illustrative study by Xing et al. [55] showed that calprotectin has a similar and remarkably high diagnostic accuracy in identifying PJI. The study reported a combined sensitivity of 0.94 (95%CI, 0.87–0.98) and specificity of 0.93 (95%CI, 0.87–0.96). The combined positive likelihood ratio (LR) was 13.65 (95%CI, 6.89–27.08) and the combined negative LR was 0.06 (95%CI, 0.02–0.15), with an AUC of 0.98 (95%CI, 0.96–0.99). This outcome surpasses commonly utilized biomarkers, offering a potential alternative for diagnosing PJI. The efficacy of clinical diagnostic indicators is typically assessed using likelihood ratio (LR) and diagnostic odds ratio (DOR). In the guide, an LR+ value greater than 5, an LR− value less than 0.2, or a DOR value greater than 10 are considered to be good predictive values. On the other hand, an LR+ value greater than 2, an LR− value less than 5, or a DOR value greater than 4 are considered to be possible predictive values (Table 3) [56, 57]. Therefore, CLP is a more effective indicator for the diagnosis of PJI, regardless of whether LR or DOR is used as the reference parameter.

**Table 3:** Summary of published studies that evaluated the diagnostic accuracy of synovial calprotectin in PJI.

Study	Warren et al. [53]	Lazic et al. [54]	Xing et al. [55]	Trotter et al. [52]
Purpose	Use multiple criteria to compare calprotectin lateral flow POC test diagnosis accuracy in TKA patients	Determine calprotectin’s accuracy in diagnosing PJI with inflammation	Study synovial Calprotectin as a PJI diagnostic test	Test performance of rapid assay for diagnosing PJI using synovial fluid calprotectin
Methods	Prospective collection of intraoperative synovial fluid samples from revision TKA patients, tested using calprotectin lateral flow POC assay	Prospective study analyzing synovial WBC, PMC, serum CRP, and synovial calprotectin in patients undergoing primary and revision THA/TKA	Meta-analysis of studies evaluating synovial Calprotectin for PJI diagnosis, adherence to PRISMA guidelines	Comparison of revision patient synovial fluid samples and lateral flow assay calprotectin measurements to ICM 2018 criteria and clinical case review gold standards
Results	Excellent sensitivity and specificity across different criteria sets, best performance with 2018 ICM criteria	Calprotectin showed good sensitivity and specificity even in cases with accompanying inflammation	High sensitivity (92%) and specificity (93%) of synovial Calprotectin for PJI diagnosis, along with high diagnostic odds ratio	Calprotectin lateral flow assay had moderate ICM criteria accuracy and improved ICM-CR gold standard accuracy
Conclusion	Calprotectin lateral flow POC test is highly sensitive and specific for diagnosing PJI in TKA patients	Calprotectin is reliable for PJI diagnosis even in cases with accompanying inflammation	Synovial Calprotectin is both cost-effective and comparable to other biomarkers for PJI diagnosis	Calprotectin lateral flow assay could be effective for diagnosing PJI, but further prospective studies are needed
Pros	Excellent sensitivity and specificity. Potential for improved diagnostic accuracy with 2018 ICM criteria. Point-of-care testing	Good sensitivity and specificity even in cases with accompanying inflammation. Reliability in primary and revision THA/TKA. Promising biomarker for PJI diagnosis	High sensitivity and specificity of synovial Calprotectin. Cost-effective and rapid diagnosis. Comparable to other biomarkers	Potential as a rapid diagnostic tool for PJI. Improvement in accuracy with clinical case review gold standard
Cons	Limited sample size. Reliance on retrospective analysis for some criteria sets	Small sample size. Lack of comparison with other biomarkers	Limited to studies using synovial Calprotectin. Potential publication bias in meta-analysis	Average accuracy compared to gold standards. Validation requires more prospective studies

PPV: positive prognostic value; NPV: negative prognostic value; AUC: area under the ROC curve.

Another commonly employed parameter in diagnostic tests is the post-test probability. This parameter indicates the probability of a patient having a PJI based on the test result being either negative or positive. The Fagan diagram demonstrates the exceptional capability of calprotectin in differentiating PJI. It seems that there is a certain level of diversity in the studies mentioned above and thus the researchers conducted a thorough subgroup analysis to identify the origin of the variation. The subgroup analyses indicated that the heterogeneity observed may be attributed to variations in the study type and detection method. Furthermore, the heterogeneity significantly decreased upon exclusion of the retrospective study.

The retrospective study conducted by Trotter et al. [52] is the sole study in the literature that examines the diagnosis of PJI. Its overall accuracy in diagnosing PJI was 75.36%, which is lower than the accuracy reported in other studies. The authors propose that the utilization of frozen storage samples may result in the breakdown of white blood cells and an increase in calprotectin levels during the process of freezing and thawing. However, there is currently a lack of recent research on this topic. The subgroup analysis results indicate that the heterogeneity in significance across studies can be attributed to the use of different tests. The calprotectin measurement methods used in these studies were lateral flow assay or ELISA. The subgroup analysis results indicate that the lateral flow assay had lower diagnostic accuracy for PJI.

## C-reactive protein

C-reactive protein (CRP) is currently measured in the serum as a widely used and cost-effective test for detecting the presence of PJI [58]. Nevertheless, the concentration of serum CRP is not specific enough to diagnose localized infection due to its presence in various noninfectious inflammatory processes as an acute-phase reactant [58]. Recent research indicates that measuring CRP in synovial fluid can be a straightforward and cost-effective way to enhance the diagnosis of PJI. This is because local CRP is believed to stimulate complement activation and phagocytosis [59]. Nevertheless, previous investigations on synovial CRP have been constrained by small sample sizes, primarily focusing on knees, and the unavailability of assays in all centers.

Synovial CRP has been investigated as a potential diagnostic tool for differentiating between inflammatory and non-inflammatory arthritis in the knee [60]. In their study, Zamani et al. [61] examined the synovial CRP levels and discovered that the synovial CRP assay effectively differentiated between osteoarthritis and inflammatory arthritis, including rheumatoid arthritis, crystal-induced arthritis, and septic arthritis. No statistically significant differences were found in the synovial CRP level between patients with inflammatory arthritis and patients with septic arthritis.

Production of C-reactive protein has also been observed in other locations, including the kidney [62], and respiratory tract [63], as well as in other tissues such as adipocytes [64] and neurons [65]. CRP was initially identified by Tillett and Francis [66] in 1930 as a component in the blood of individuals with acute inflammation that interacted with the C polysaccharide of pneumococcus. The condition exhibits rapid onset and peak, and promptly subsides following the injury. CRP specifically attaches to phosphocholine molecules found in microorganisms. CRP is believed to aid in the activation of the complement system and improve the process of phagocytosis by macrophages, which have a receptor for CRP [59].

Serum CRP has shown its value as a diagnostic test for PJI. Ghanem et al. [58] conducted a study to assess the accuracy of serum ESR and CRP in detecting PJI. They observed that when both tests were used together, with cutoff values of 30 mm/h for ESR and 20.5 mg/L for CRP, they achieved a sensitivity of 96% and a specificity of 59%. Greidanus et al. [67] reported comparable findings with a higher level of specificity. Out of the 151 knees that underwent revision TKA, a diagnosis of PJI was confirmed for 45 of them. The receiver-operating-characteristic curves showed that the most effective threshold for positivity was 22.5 mm/h for the ESR and 13.5 mg/L for the CRP levels. The ESR (sensitivity, 0.93; specificity, 0.83; positive likelihood ratio, 5.81; accuracy, 0.86) and the CRP level (sensitivity, 0.91; specificity, 0.86; positive likelihood ratio, 6.89; accuracy, 0.88) both demonstrate outstanding diagnostic test performance.

The measurement of CRP is already a standard clinical test that can be conducted quickly and inexpensively. In 2012, Parvizi et al. [68] conducted their study using standard laboratory equipment to measure serum CRP levels. This laboratory test is efficient and affordable, taking approximately one hour to complete and costing around $17. Their research shows that measuring CRP levels in synovial fluid, rather than in the serum, improves the diagnostic accuracy for PJI. Additionally, a strong correlation was discovered between the levels of CRP in the blood serum and the levels of CRP in the synovial fluid (*r*^2^ = 0.72).

Catterall et al. [69] reported a correlation between the levels of CRP in the synovial fluid and the levels of CRP in the blood serum of patients with acute knee trauma. The researchers hypothesized that the movement of serum CRP into the joint could be the cause of the increased levels of CRP in the synovial fluid, particularly when the serum CRP levels were also elevated. This mechanism may also be applicable to patients with PJI. During an infection, inflammation in the synovial membrane can increase its permeability, allowing high levels of serum CRP to enter the joint and raise the levels of CRP in the synovial fluid. Similarly, Wang et al. [70] conducted a meta-analysis of 6 studies that showed that synovial fluid CRP is an effective biomarker for the diagnosis of PJI, with a high level of sensitivity (0.92) and specificity (0.90) (Table 4).

**Table 4:** The diagnostic accuracy of CRP as biomarker in PJI.

Study	Ghanem et al. [58]	Greidanus et al. [67]	Parvizi et al. [68]	Wang et al. [70]
Aim	Assess the efficacy of CRP monitoring in diagnosing persistent PJI	Evaluate diagnostic test characteristics of ESR and CRP for PJI diagnosis	Examine synovial CRP quantification for PJI diagnosis	Evaluate synovial fluid CRP as a biomarker for PJI diagnosis
methods	Retrospective analysis of arthroplasty database, ROC curve analysis	Prospective evaluation of patients for infection with measurement of ESR and CRP levels	Prospective collection of synovial fluid samples, comparison between septic and aseptic groups	Systematic review and meta-analysis of studies assessing CRP in PJI diagnosis
Results	Treatment group mean CRP values were not statistically different. AUCs were 0.46–0.73	Specificity, sensitivity, and positive likelihood ratio make ESR and CRP good diagnostic tools	Statistically significant difference in synovial CRP between septic and aseptic groups	Combined sensitivity, specificity, diagnostic odds ratio, and AUSROC are diagnostically valuable
Conclusions	CRP monitoring does not indicate successful eradication of PJI	ESR and CRP provide excellent diagnostic information for PJI diagnosis	Synovial CRP assay holds promise as a diagnostic marker for PJI	Synovial fluid CRP is a good biomarker for PJI diagnosis
Pros	Large sample size, retrospective analysis	Prospective design, assessment of multiple diagnostic parameters	Prospective design, direct comparison between septic and aseptic groups	Meta-analysis provides comprehensive overview, high sensitivity and specificity
Cons	Lack of prospective design, limited to CRP monitoring	Patient selection may be biased, limited to revision total knee arthroplasty patients	Limited to synovial CRP quantification, potential variability in synovial fluid collection and analysis	Limited to studies assessing CRP, potential heterogeneity among included studies

In 2023, Grzelecki et al. [71] conducted a study to assess the diagnostic accuracy of various POCTs in detecting CRP in synovial fluid for the diagnosis of PJI. Synovial fluid samples were obtained from 120 consecutive patients who underwent revision TJA. The individuals were categorized into two distinct groups. The initial cohort consisted of 76 patients who underwent revision surgery for non-infectious reasons, referred to as the aseptic revision TJA group. The second cohort comprised 44 patients who underwent revision surgery specifically due to PJI. Four rapid CRP tests with varying threshold values (1 and 3 mg/L, ≥8 mg/L, ≥10 mg/L [cassette], ≥10 mg/L [strip]) were utilized for synovial fluid testing, despite not being officially approved for this purpose. Tests were conducted on identical synovial fluid samples, and the findings of these tests were compared to those obtained using the laboratory technique. The cassette test, with a minimum cutoff value of ≥8 mg/L showed the highest accuracy in the diagnosis of chronic PJI, with a sensitivity of 90.9% and a specificity of 90.8%. The cassette test, with a cutoff value of >3 mg/L showed a sensitivity of 68.2% and a specificity of 77.6%. The sensitivity and specificity of the cassette test were 77.3% and 94.7%, respectively, for tests with a minimum cutoff value of ≥10 mg/L. For the strip test, the sensitivity and specificity were 77.3% and 96.1%, respectively. The laboratory method utilizing a statistically determined threshold of 2.7 mg/L showed the highest AUC value of 0.95, along with a sensitivity of 90.9% and specificity of 94.7% (Table 5).

**Table 5:** Comparison of sensitivity and specificity between several CRP POCT and the classic CRP laboratory examination.

POCT	Sensitivity (%)	Specificity (%)
Cassette (≥8 mg/L cutoff)	90.9	90.38
Cassette (>3 mg/L cutoff)	68.2	77.6
Cassette (≥10 mg/L cutoff)	77.3	94.7
Strip	77.3	96.1
Laboratory method (threshold of 2.7 mg/L)	90.9	94.7

## Conclusion

POCTs are useful for the diagnosis of PJI due to their convenience and accessibility. From ward glucose meters to LE strip tests and calprotectin lateral flow assays, these tests help clinicians detect PJI quickly. Despite their usefulness, accuracy and reliability issues remain. POCT requires strict quality control, often requiring manufacturer-supplied calibrators and materials. Calibration using external factors raises concerns about consistency and accuracy, especially when administered by non-laboratory staff. Advances in automation, quantification, and artificial intelligence (AI) offer hope for overcoming these challenges. AI algorithms could improve POCT accuracy and precision, revolutionizing PJI diagnosis. Novel tests such as the calprotectin lateral flow assay outperform traditional biomarkers such as white blood cell count, PMN %, and CRP.

POCT is a major advance in PJI diagnosis, but more research is needed to improve their efficacy. Larger prospective studies are needed to determine these tests’ clinical utility. Clinicians can improve patient outcomes and quality of care by navigating POCT and using emerging technologies to diagnose PJI more accurately and quickly.

## Data Availability

Data are available on request from the authors.
